# Nurse staffing and inpatient mortality in the English National Health Service: a retrospective longitudinal study

**DOI:** 10.1136/bmjqs-2022-015291

**Published:** 2022-09-27

**Authors:** Ben Zaranko, Natalie Jean Sanford, Elaine Kelly, Anne Marie Rafferty, James Bird, Luca Mercuri, Janice Sigsworth, Mary Wells, Carol Propper

**Affiliations:** 1 Institute for Fiscal Studies, London, UK; 2 Florence Nightingale Faculty of Nursing Midwifery and Palliative Care, King's College London, London, UK; 3 The Health Foundation, London, UK; 4 Imperial College Healthcare NHS Trust, London, UK; 5 Research Informatics Team, Imperial College Healthcare NHS Trust, London, UK; 6 Department of Economics and Public Policy, Imperial College Business School, London, UK

**Keywords:** health policy, nurses, duty hours/work hours, teams

## Abstract

**Objective:**

To examine the impact of nursing team size and composition on inpatient hospital mortality.

**Design:**

A retrospective longitudinal study using linked nursing staff rostering and patient data. Multilevel conditional logistic regression models with adjustment for patient characteristics, day and time-invariant ward differences estimated the association between inpatient mortality and staffing at the ward-day level. Two staffing measures were constructed: the fraction of target hours worked (fill-rate) and the absolute difference from target hours.

**Setting:**

Three hospitals within a single National Health Service Trust in England.

**Participants:**

19 287 ward-day observations with information on 4498 nurses and 66 923 hospital admissions in 53 inpatient hospital wards for acutely ill adult patients for calendar year 2017.

**Main outcome measure:**

In-hospital deaths.

**Results:**

A statistically significant association between the fill-rate for registered nurses (RNs) and inpatient mortality (OR 0.9883, 95% CI 0.9773 to 0.9996, p=0.0416) was found only for RNs hospital employees. There was no association for healthcare support workers (HCSWs) or agency workers. On average, an extra 12-hour shift by an RN was associated with a reduction in the odds of a patient death of 9.6% (OR 0.9044, 95% CI 0.8219 to 0.9966, p=0.0416). An additional senior RN (in NHS pay band 7 or 8) had 2.2 times the impact of an additional band 5 RN (fill-rate for bands 7 and 8: OR 0.9760, 95% CI 0.9551 to 0.9973, p=0.0275; band 5: OR 0.9893, 95% CI 0.9771 to 1.0017, p=0.0907).

**Conclusions:**

RN staffing and seniority levels were associated with patient mortality. The lack of association for HCSWs and agency nurses indicates they are not effective substitutes for RNs who regularly work on the ward.

**WHAT IS ALREADY KNOWN ON THIS TOPIC**

- Previous research has established that higher levels of nurse staffing improve patient outcomes, with analysis typically conducted at the level of either the patient or hospital.
- Far less is understood about the role of the composition of the nursing team in determining patient outcomes, despite the centrality of teamwork to the delivery of healthcare.
- A small number of previous studies have distinguished between registered nurses (RNs) and healthcare support workers (HCSWs), but none has distinguished between RNs of different seniority levels or contract types, and many rely on cross-sectional study designs which cannot convincingly demonstrate causality

**WHAT THIS STUDY ADDS**

- Our study shows that both the level of RN staffing and the seniority mix of RNs were associated with patient mortality outcomes, but HCSW and agency nurse staffing were not.
- We present new evidence of non-linear impacts of deviations from target staffing, with statistically significant impacts of below-target RN staffing only appearing when the team was short-staffed by more than one team member.
- Our empirical approach makes a causal interpretation of our results more plausible than in many previous studies.

HOW THIS STUDY MIGHT AFFECT RESEARCH, PRACTICE OR POLICY

- Our study suggests that policy should focus on increasing the number of trained RNs, fostering professional growth and progression for RNs, and improving the retention of existing staff in clinical practice roles.
- Our focus on the ward-day (team) level provides evidence with direct relevance for workforce planning.

## Introduction

Teams of nursing staff play a critical role in healthcare delivery. Identifying strategies to optimise the staffing of these teams is a priority for health service providers and policymakers. A growing body of research suggests that a richer nursing skill-mix, greater education levels and higher nurse-to-patient ratios are associated with better patient outcomes, increased staff well-being, decreased healthcare spending and improved workforce retention.^1–11^ Patient outcomes that have been examined include mortality,^8 11–17^ missed care^13 18 19^ and nurse-driven outcomes like pressure injuries, falls and medication errors.^14 17 20–22^ While there is consensus that improving nurse staffing improves patient outcomes, the role of the nursing team and the impact of its size and composition on outcomes remains relatively unexplored. Human capital theory suggests that team composition, in addition to size, matters. Becker^23^ distinguishes between general and firm-specific human capital. The former is derived from higher skills or qualifications. The latter is built up from the workers’ familiarity with their physical environment and co-workers. Teams are composed of individuals with different levels of general and firm-specific human capital and these factors, in addition to team size, will influence outcomes.

Exploration of the role of different types of human capital has been undertaken in various settings outside^24 25^ and inside^26–28^ healthcare. Related research has examined skill-mix, an element of human capital, in the nursing context.^27–32^ This has typically been done by distinguishing between two groups: registered nurses (RNs) and healthcare support workers (HCSWs). RNs are fully qualified nurses on the Nursing and Midwifery Council register, who have completed formal training and typically hold a university diploma or degree-level qualification. HCSWs are personnel who report to RNs and support the delivery of nursing care, but do not have professional registration. Griffiths *et al* 30 used routine data from an e-rostering system to examine RN and HCSW staff levels on patient mortality, controlling for confounders such as patient acuity, patient turnover and ward-level factors. The authors found that the risk of death increased when either RN or HCSW levels fell below the ward mean. There was also some indication that patient risk of death increased when HCSW levels were *above* the ward mean. This may either suggest that diluting the skill-mix by incorporating additional HCSWs can have negative impacts on patient mortality, or it may indicate that extra HCSWs are brought in when patients are sicker. In a related study, Smith *et al* 29 examined a mechanism through which nurse staffing levels affect patient outcomes. The authors found that RN, but not HCSW, staffing levels affected the rates of failure to respond to the deterioration of the most acutely ill patients. Dall’Orra *et al* 33 examined the role of temporary nurse staffing and patient mortality and found that heavy reliance on temporary staff—who are likely to have less firm-specific capital—was associated with a higher risk of a patient death.

While these studies provide some insight into how human capital impacts patient outcomes, they are at the patient level and do not focus explicitly on the role of team production. As such, they do not directly examine the effect of the nursing team composition, and do not focus on how different combinations of nurses, with different levels of general or firm-specific capital impact team performance (as measured by patient outcomes). This limits the applicability of the results in two ways. First, previous papers have not distinguished between RNs of different levels of seniority, nor between staff who are directly employed by the hospital and those on agency contracts. Second, nurse staffing is planned in advance at the team level, whereas most existing studies are at the patient or hospital level and often use staffing measures that are only available after the fact (such as nurse-reported staffing levels or care hours per patient). This limits their applicability for understanding how the make-up of the nursing teams impacts patient outcomes and for improving workforce planning by managers.

In order to optimise, managers need to understand the relative improvements in patient outcomes that come from adding a less qualified individual or one less familiar with the hospital/ward compared with a more qualified individual with greater ward-specific and team-specific experience. To address this gap, this paper provides the results of a retrospective panel study which links existing routine data from electronic staff rosters (ESRs) and patient records to examine the impact of nursing team size and composition on patient mortality in the hospital setting.

## Methods

### Study aims

1. To examine the impact of nursing team staffing on inpatient mortality after accounting for differences in hospital, ward and patient characteristics.
2. To determine whether these effects vary by the general human capital (skill level) of the team, by comparing the effect of an increase in HCSWs with an increase in RNs of different seniority levels.
3. To ascertain whether these effects are impacted by firm-specific human capital, by comparing the impact of an increase in staff with different levels of experience in the ward and hospital studied.
4. To establish whether there is a threshold effect on inpatient mortality when actual nurse staffing levels are a certain level below target staffing.

### Study design

This study leveraged a novel linkage between ESR and electronic patient records (EMR) data for three hospitals within a large English National Health Service (NHS) Hospital Trust in 2017. The Trust had 98 ‘staffing units’ (generally comprising one but sometimes two wards, referred to henceforth as wards for simplicity). The analysis focused on inpatient wards responsible for treating acutely ill adult patients. Maternity and paediatric wards, wards that regularly closed (having a day with zero patients 30 times or more in the calendar year), wards that employed no RNs and wards that had zero patient deaths over the calendar year were excluded from the study.

**Figure 1:**
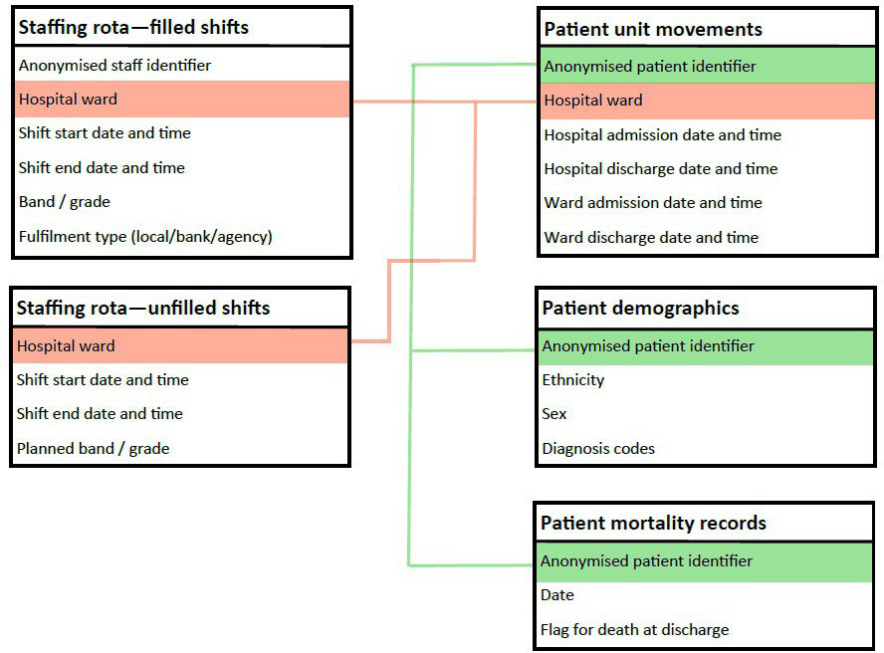
Data structure.

The ESR data provided detailed information on each shift worked by nursing staff in the NHS Trust including the hospital ward, date, start and end time, NHS Agenda for Change pay band (a measure of seniority and whether the staff member is a RN) and contract type (permanent, bank or agency). Patient-level EMR data provided information on when and in which ward(s) patients were treated and detailed information on patient demographics, diagnoses (with coding from the International Classification of Diseases, 10th edition (ICD-10)),^34^ discharge status and whether the patient died in hospital (see figure 1). We define each ward-day to be a ‘team’ as this is the broad level at which staffing planning is done, and the 24-hour continuum is highly relevant for care delivery. Patient-level and staffing-level data were linked at the ward-day (24 hours) level for the analysis.

## Measures

### Staffing

In the ESR, shifts are rostered at the ward level at least 8 weeks in advance (the planned staffing). Rostered shifts are not always filled (the actual staffing) because of absences (planned and unplanned) or vacancies. The system records information on shifts that were not filled, including the shift timing, duration and planned band level of the unfilled staff member. The measures used in the analysis were derived from the ‘fill-rate’: the proportion of planned staff hours that were worked. A fill-rate of <100% indicates that some planned staff hours on that day went ‘unfilled’.

There were two broad skill groups: RNs and HCSWs. RNs were identified in our data as those in NHS Agenda for Change (AfC) pay band 5 and above and HCSWs as those in AfC pay bands 2–4. The study was conducted before the introduction of nursing associate roles. Students were considered supernumerary and were excluded.

To measure the quantity of staff, the proportion of hours filled by RNs and HCSWs was calculated. This was further split by pay band within RNs. This enabled us to capture the seniority and skill-mix, and so the general human capital, of the team. To examine firm-specific capital, we distinguished between the proportions of planned hours filled by those employed by the NHS Trust working in their regular ward (permanent staff), by those employed by the Trust doing extracontractual work (bank staff) and by those contracted to work temporarily in the Trust (agency staff). To examine non-linear impacts of having below planned levels of staffing, six separate binary indicators were defined and coded as 1 if team staffing was more than 4, 8, 12, 16, 20 or 24 hours below the planned staffing level, respectively. For example, a team 14 hours short of the planned hours would have the first three of these variables coded as 1 and the latter three as 0. This was constructed separately for HCSWs and RNs. The fill-rate and binary measures are related, but while the fill-rate adjusts for the size of the ward in question and thus provides a measure of the relative deviation from planned staffing, the absolute deviation from target hours does not.

### Patient outcome

The patient outcome measure used was inpatient mortality at discharge. This measure was selected because it is unambiguous, consistently recorded across wards within the Trust and used in previous similar research.^11 16 30^ A binary indicator was coded as 1 if the team (ward-day) had at least one death, and zero otherwise.

### Patient characteristics

The number of patients, mean age, gender, the proportion of patients recorded as non-white (ethnicity recorded as ‘unknown’ coded as non-white), mean Elixhauser Comorbidity Index score^35^ and fraction of patients with primary diagnosis in each ICD-10 chapter were constructed at the ward-day level and linked to ward-day staffing measures. All mean patient characteristics were constructed by weighting patients by the hours spent in the ward.

### Statistical analysis

The characteristics of patients and nursing teams were summarised by their means and SD. Eleven separate conditional regression models of patient mortality were estimated to address the study aims. The first examined the impact of nurse quantity using the overall fill-rate. The second examined the effect of different general human capital levels and compared the impacts of the fill-rate for RNs and HCSWs separately. The third examined whether, within the category of RNs, the impacts varied by seniority by separating NHS AfC bands 5, 6 and 7–8. The fourth and fifth examined hospital and team familiarity by examining whether the impact of the fill-rate for RNs and nursing support staff, respectively, varied by contract type. The final six models examined whether effects emerged only when actual nurse staffing levels were a certain amount below target staffing and whether these effects were non-linear. They separately examined the impact of being 4, 8, 12, 16, 20 and 24 hours or more below target staffing hours, respectively.

All models were estimated as multilevel conditional logistic regressions, also known as a fixed-effects logit model, at the ward-day level. ORs were calculated for patient mortality. The regressions controlled for hospital ward fixed effects to capture average and therefore time-invariant differences in the odds of a death by ward across the whole year. Factors that might contribute to these ward-level differences include medical specialty, average patient mix, average staffing levels and physical layout. The regressions also include time-varying patient characteristics (such as age, gender and ethnicity), the number of patients treated and time variables (day of the week, month and public holidays). Models were estimated with the mean Elixhauser Comorbidity Index and, separately, with the fraction of patients with a primary diagnosis in each ICD-10 chapter. The results were close to identical and so, in favour of a more parsimonious model with greater df, only the mean Elixhauser Comorbidity Index was included in the final specification; 95% CIs were calculated. The inclusion of ward fixed effects meant the analysis compared days when a ward had higher staffing levels with other days when the same ward had lower staffing levels. Therefore, the OR estimates show the change in the probability of a patient death associated with a change in our staffing measures. SEs were clustered at the hospital ward level. All statistical analyses were conducted using the *clogit* command in Stata V.17.

### Data anonymisation and access

The study used retrospective anonymised extracts of routinely captured clinical information and staffing data. Patient-identifiable data were not used. Anonymised data were accessed with explicit permission from the NHS Data Protection Office and Caldicott Guardian and followed the protocols for data access and analysis used in the Trust.

### Patient and public involvement

Patients and/or the public were not involved in the design, or conduct, or reporting, or dissemination plans of this research.

## Results

### Characteristics of teams: staffing, patients and patient outcomes

The data contained information on 297 654 shifts worked by 4498 unique members of staff, 44 634 unique patients with 66 923 hospital spells (ie, 66 923 separate admissions to, and periods of treatment, in hospital) and 53 wards. After excluding 58 ward-days without any patients, the final sample had 19 287 ward-day observations (and so 19 287 teams). The average number of rostered staffing hours per ward-day was 193.4 (SD 89.6), of which an average of 52.9 hours were rostered for HCSWs (SD 30.6) and 140.5 hours for RNs (SD 90.8) (table 1). This is equivalent to a mean of 16 staff members per ward working 12-hour shifts, with 4 HCSWs and 12 RNs in the average team (with variation across wards).

**Table 1:** Staffing levels in sample hospital wards during analysis period

	All nursing staff		Healthcare support workers		Registered nurses	
	Mean	SD	Mean	SD	Mean	SD
Rostered hours	193.4	89.6	52.9	30.6	140.5	90.8
Actual hours worked	185.4	88.5	49.8	29.0	135.7	90.0
Unfilled hours	7.9	10.0	3.1	6.1	4.8	7.6
Rostered hours per patient day	15.2	14.8	3.7	3.9	11.5	13.3
Hours worked per patient day	14.6	14.3	3.5	3.8	11.1	13.0
Unfilled hours per patient day	0.6	1.3	0.2	0.6	0.4	1.1
Fill-rate, of which:	95.7	5.4	94.6	11.2	96.4	5.8
Local/Regular staff (%)	68.3	15.8	64.3	26.5	70.8	16.9
Bank staff (%)	20.4	12.7	28.3	23.9	16.2	12.7
Agency staff (%)	7.1	8.2	1.9	8.3	9.4	11.0
Band 2 staff (%)	23.8	14.5	77.7	25.6	–	–
Band 3 staff (%)	3.8	5.5	16.0	23.7	–	–
Band 4 staff (%)	0.2	1.0	0.9	3.7	–	–
Band 5 staff (%)	47.2	11.5	–	–	68.4	15.7
Band 6 staff (%)	17.0	12.4	–	–	22.8	13.3
Bands 7 and 8 staff (%)	3.8	4.5	–	–	5.2	5.8
Staffing below target by at least:						
4 hours	0.51	0.50	0.25	0.44	0.36	0.48
8 hours	0.47	0.50	0.23	0.42	0.33	0.47
12 hours	0.36	0.48	0.17	0.37	0.22	0.41
16 hours	0.20	0.40	0.06	0.23	0.10	0.30
20 hours	0.15	0.36	0.03	0.18	0.07	0.25
24 hours	0.10	0.29	0.01	0.11	0.03	0.18
Observations	19 287		19 287		19 287	
Hospital wards	53		53		53	

Unit of analysis is the hospital ward-day. Fill-rate is defined as the number of actual hours worked divided by the number of rostered hours. The fill-rate for local, bank and agency staff, and for each NHS pay band, is calculated as the number of hours worked by staff of that type divided by the number of rostered hours. Target staffing is defined as the number of rostered hours in a given ward on a given day. Hours per patient day is defined as the number of staff hours divided by the patient census at midnight (as per the English NHS definition of care hours per patient day).

NHS, National Health Service.

In 51% of teams, the actual number of hours worked was less than the number of rostered hours. The average fill-rate was 95.7% (SD 5.4). This was slightly lower for HCSWs (94.6%, SD 11.2) than for RNs (96.4%, SD 5.8); 68.3% (SD 15.8) of rostered hours were worked by permanent staff, 20.4% (SD 12.7) were worked by bank staff and 7.1% (SD 8.2) by agency workers. Thirty-six per cent of teams were 4 hours or more below target RN staffing; 33% were 8 hours or more below; 22%, 12 hours; 10%, 16 hours; 7%, 20 hours and 3% were 24 hours or more below target RN staffing, respectively.

One thousand one hundred ninety-eight (6.2%) of the teams had a patient death (table 2). Equivalently, on 6.2% of ward-days, at least one patient died. The average team treated 20.6 patients (SD 8.4), with a mean age of 64.1 (SD 9.8) and mean Elixhauser Comorbidity Index of 3.7 (SD 1.2).

**Table 2:** Patient outcomes and characteristics in sample hospital wards during analysis period

	Mean	SD
Number of patients treated in the ward	20.63	8.39
Age	64.14	9.78
Female (%)	0.46	0.18
Non-white (%)*	0.52	0.16
Elixhauser Comorbidity Index score	3.68	1.16
Average length of hospital stay (days)†	28.35	16.99
In-hospital patient death (binary)	0.062	0.241
Death rate per 1000 patients	3.51	16.06
Observations	19 287	
Hospital wards	53	

Unit of analysis is the hospital ward-day. Patient characteristics are weighted by the hours spent in the ward on the day in question.

*Non-white includes patients for whom ethnicity was recorded as unknown.

†Average length of stay is calculated here as the mean length of hospital stay among the patients treated by each team (ie, on each ward-day), in order to capture (one aspect of) the severity of those patients. A patient who stays in one hospital ward hospital for 10 days, for example, would be treated by 10 teams, and so their length of stay (10 days) would enter the mean value for 10 separate teams (appropriately, given that the patient’s severity affects the workload for each of those teams). In contrast, a patient who stayed in hospital for <1 day, under the care of a single team, would only have their length of stay (1 day) enter the mean value for one team. For this reason, patients with long stays are over-represented, which acts to increase the overall mean and this measure of average length of stay (with respect to the team) is therefore higher than and not comparable to the average length of stay for a patient.

### Association between staffing levels and inpatient mortality

#### The effect of general and firm-specific capital

There was no statistically significant association between the overall fill-rate and the odds of patient death (OR 0.9934, 95% CI 0.9794 to 1.0076, p=0.3605) (table 3). Splitting by staffing group (a proxy for skill level or general human capital), a percentage point increase in the fill-rate for RNs was associated with a reduction in the odds of patient death by around 1.2% (OR 0.9883, 95% CI 0.9773 to 0.9996, p=0.0416). The average team had 140.5 rostered RN hours, meaning that a 12-hour increase in RN staffing is equivalent to an average increase in the fill-rate of 8.5 percentage points. These estimates therefore imply that, for the average team, an extra 12-hour shift from an RN would reduce the odds of experiencing a patient death by around 9.6% (OR 0.9044, 95% CI 0.8219 to 0.9966, p=0.0416). The fill-rate for HCSWs had no significant effect on patient mortality.

**Table 3:** Adjusted ORs indicating the change in the odds of a patient death following a change in the nursing staff fill-rate

	Outcome: patient death		
	OR	95% CI	P value
Fill-rate (ppt) for all staff	0.9934	0.9794 to 1.0076	0.3605
Fill-rate (ppt) for:			
Healthcare support workers (HCSWs)	1.0029	0.9964 to 1.0095	0.3748
Registered nurses (RNs)	0.9883	0.9773 to 0.9996	0.0416
Share (ppt) of rostered hours for HCSWs worked by:			
Local/Regular	1.0028	0.9961 to 1.0095	0.4167
Bank	1.0030	0.9963 to 1.0097	0.3834
Agency	1.0021	0.9907 to 1.0137	0.7208
Share (ppt) of rostered hours for RNs worked by:			
Local/Regular	0.9875	0.9757 to 0.9994	0.0389
Bank	0.9861	0.9729 to 0.9995	0.0427
Agency	0.9913	0.9797 to 1.0031	0.1464
Share (ppt) of rostered hours for RNs worked by:			
Band 5	0.9893	0.9771 to 1.0017	0.0907
Band 6	0.9845	0.9714 to 0.9977	0.0214
Bands 7 and 8	0.9760	0.9551 to 0.9973	0.0275

Results listed are from five separate conditional logistic regression models, each with ward fixed effects. Unit of analysis is the hospital ward-day (n=19 287). HCSWs, defined as those in NHS pay bands 2–4. RNs, defined as those in NHS pay band 5 and above. 95% CI, with SEs clustered at the hospital ward level. All models also control for the mean patient age; the patient age squared; the sex and ethnicity mix of patients; the mean Elixhauser Comorbidity Index; pairwise-interaction terms between mean age, sex, ethnicity and Elixhauser Comorbidity Index; the mean hospital length of stay of patients treated in the ward that day; the number of patients treated in the ward that day; a dummy for each day of the week; a dummy for month of the year and a dummy indicating whether the day was a bank holiday. Fill-rate is defined as the number of actual hours worked divided by the number of planned (rostered) hours. The fill-rate for healthcare support workers and registered nurses is calculated relative to the planned hours for staff of that type.

NHS, National Health Service; ppt, percentage point.

Further analysis of the impact of general human capital (or skill level, as measured by staffing band) shows that an extra one percentage point of planned RN hours filled by a band 5 nurse was associated with a reduction in the odds of patient death by 1.1% (OR 0.9893, 95% CI 0.9771 to 1.0017, p=0.0907); for a band 6 nurse, the associated reduction was 1.6% (OR 0.9845, 95% CI 0.9714 to 0.9977, p=0.0214) and for bands 7–8 nurses, 2.4% (OR 0.9760, 95% CI 0.9551 to 0.9973, p=0.0275).

When evaluating firm-specific capital, a one percentage point increase in the proportion of rostered RN hours worked by permanent and bank RNs was associated with a statistically significant reduction of 1.2%–1.4% in the odds of patient death (local: OR 0.9875, 95% CI 0.9757 to 0.9994, p=0.0389; bank: OR 0.9861, 95% CI 0.9729 to 0.9995, p=0.0427). The OR for agency nurses was smaller and not statistically different from zero (OR 0.9913, 95% CI 0.9797 to 1.0031, p=0.1464). The effects of the proportion of rostered hours filled by permanent, bank and agency among nursing support staff were not significantly different from zero (table 3).

#### Below-target staffing

Teams with RN staffing 16 hours or more below target had 22.8% higher odds of patient death (OR 1.2282, 95% CI 1.0034 to 1.5035, p=0.0463), rising to 26.3% higher for teams with RN staffing 20 hours or more below target (OR 1.2626, 95% CI 0.9841 to 0.6199, p=0.0647) and 37.0% higher for teams 24 hours or more below target (OR 1.3701, 95% CI 1.0146 to 1.8501, p=0.0399) (table 4). The results for RN staffing 4, 8 and 12 hours below target were not statistically significant. In supplementary analyses, no statistically significant impacts of below-target staffing of HCSWs were found (available on request).

**Table 4:** Adjusted ORs indicating the change in the odds of a patient death associated with below-target registered nurse (RN) staffing

	Outcome: patient death		
	OR	95% CI	P value
Binary indicator of RN staffing below target by:			
4 hours or more	1.0424	0.9257 to 1.1739	0.4927
8 hours or more	1.0587	0.9371 to 1.1961	0.3596
12 hours or more	1.0933	0.9508 to 1.2572	0.2108
16 hours or more	1.2282	1.0034 to 1.5035	0.0463
20 hours or more	1.2626	0.9841 to 1.6199	0.0667
24 hours or more	1.3701	1.0146 to 1.8501	0.0399

Results listed are from six separate conditional logistic regression models, each with ward fixed effects. Unit of analysis is the hospital ward-day (n=19 287). 95% CI, with SEs clustered at the hospital ward level. All models also control for the mean patient age; the patient age squared; the sex and ethnicity mix of patients; the mean Elixhauser Comorbidity Index; pairwise-interaction terms between mean age, sex, ethnicity and Elixhauser Comorbidity Index; the mean hospital length of stay of patients treated in the ward that day; the number of patients treated in the ward that day; a dummy for each day of the week; a dummy for month of the year and a dummy indicating whether the day was a bank holiday. Target RN staffing is defined as the total number of rostered hours for RNs (those in band 5 or above), which is equal to the sum of filled and unfilled hours.

## Discussion

There are four key findings of this study. First, higher general human capital is associated with better outcomes. Teams that filled a lower percentage of their rostered RN hours experienced significantly more patient deaths than teams with higher RN staffing levels. Lower levels of HCSW staffing were not associated with an increase in the odds of patient death. Second, there are significant returns to increasing seniority levels within the RNs. An additional senior nurse (NHS AfC pay band 7 or 8) was associated with more than twice the effect on lowering the odds of a patient death than an additional nurse in pay band 5, and 1.5 times the impact of an additional nurse in pay band 6. Third, firm-specific capital matters. Among RNs, only the addition of more nurses employed by the Trust (regular shifts or overtime via the bank) was associated with a reduction in the odds of a patient death; the hours supplied by agency nurses had no significant effect. Finally, the impacts of below-target RN staffing increased depending on how short-staffed the team was relative to target. The estimates indicate that patient mortality risk was positively associated with all levels of RN staffing shortfall, but the size of the effect jumped and became statistically significant only when the team was short-staffed by at least 16 hours. This is consistent with a ‘threshold effect’ where the effect of being short-staffed arises when teams are short more than one RN working a 12-hour shift.

Much of the literature showing a positive association between nurse staffing and patient outcomes uses a cross-sectional design. Such studies struggle to convincingly demonstrate causality. Many studies rely on retrospective, nurse-reported staffing levels.^2 3 5 11 13 16–18 20 22 30–32 36^ Our study uses administrative panel data to address these methodological concerns and makes several additional contributions. This is, to our knowledge, the first to approach the problem from the perspective of the nursing team. It is important to consider nurse staffing at the team level because teams are critical building blocks for the delivery of nursing care.^28 37^ We demonstrate that routinely collected ex ante measures of staffing (the fill-rate for RNs and the difference between actual and target RN staffing hours) are associated with patient mortality rates. This provides convincing evidence which is of direct relevance to workforce planning and complements previous work by Griffiths *et al* 30 that demonstrated the link between an ex post measure of staffing (care hours per patient day) and inpatient mortality. In addition, we believe this to be the first study showing differential impacts of RNs of different contract types, who differ by levels of hospital-specific and ward-specific human capital. This study is also the first to show the differential impacts of RNs by seniority. These results demonstrate the value of ensuring and retaining an adequate number of regularly employed RNs, show the significant value of senior, more experienced nurses who provide team leadership and, ultimately, highlight areas to target when mobilising extra resources.

### Strengths and limitations

Our study has several strengths. First, we were able to precisely link patients to the nursing teams responsible for their care in our administrative data, and our analysis could thus be conducted at a much more granular level than many previous studies that have been conducted using monthly or hospital-level data. Second, we employed panel data and were thus able to robustly control for time-invariant factors such as ward specialty and typical patient mix, both of which might affect patient outcomes. The study was observational, but this empirical approach makes a causal interpretation more plausible (though not certain). Third, we were able to distinguish between different staff types: RNs versus HCSWs, permanent nurses versus bank and agency nurses (extending previous work by Dall’Orra *et al*,^33^ who were unable to distinguish between bank and agency staff) and RNs of differing seniority, which has previously not been examined. Fourth, our primary staffing measure (the ‘fill-rate’) accounts for variation in ward and team size and thus captures the impacts of relative deviations in staffing from the planned level. Fifth, the granularity of our data allowed us to additionally explore possible non-linear impacts in deviations from target levels of staffing. Our evidence suggests that problems emerge when more than one RN is missing from the team and when senior, ward-based RN hours are not filled. Finally, because we conduct our analysis at the team level and employ measures of staffing available to management before outcomes are realised, we provide evidence of direct relevance for workforce planning.

There are several limitations to this study. First, while we control for observable patient severity using all the information available to us, some aspects of patient severity will remain unobservable. We do not have information on the mode of admission, for instance, which represents a limitation of our dataset relative to some other patient datasets. This introduces the possibility of omitted variable bias. It is also possible that, when patients are sicker in a way that cannot be controlled for in the research, managers take greater steps to fill rostered shifts, thereby increasing the fill-rate when the risk of patient death is higher. This simultaneity, combined with the omitted variable bias, would likely bias our estimates towards finding no effect of below-target staffing. This may explain our finding that shortages of HCSWs and agency RNs have no significant impact on the odds of patient death. It also means that our estimates of the negative impact of RN staffing on deaths may understate the ‘true’ effect. Nonetheless, our inclusion of hospital ward fixed effects and a rich array of patient characteristics goes beyond much of the previous literature in controlling for unobserved patient acuity, and the fact that we find significant negative impacts of RN staffing on the odds of patient death despite the bias in the opposite direction should provide confidence in our results. Second, we use a single outcome measure, patient mortality. Nursing staff may have significant impacts on other important outcomes not examined here. Third, we examine only contemporaneous impacts of nurse staffing on patient outcomes (ie, on the same ward-day) and so do not capture any lagged effects from nurse staffing on previous days. However, we have examined this directly, and find no evidence of such lagged effects on patients or spillovers onto other teams, suggesting that this is not a major limitation of our study. Fourth, our study was also limited to a single NHS Trust, although we found results consistent with those of Griffiths *et al*,^30^ who used administrative data from another Trust in England. Finally, our study is unable to shed light on the precise mechanisms through which changes in nurse staffing affect patient outcomes, although our evidence on the importance of the most senior nurses suggests an important role for leadership, decision-making and team management.

## Conclusion

These findings demonstrate the potential adverse consequences of nursing shortages, particularly of permanent staff. The proportion of experienced nurses who are working in their usual place of work and senior nurse managers in a nursing team are significantly associated with patient mortality. Additional HCSWs and agency RNs have no significant impact and therefore should not, at the margin, be treated as effective substitutes for experienced permanent RNs. Instead, policy should be directed towards increasing the number of trained RNs, fostering professional growth and progression for RNs and improving the retention of existing staff in clinical practice roles. Our study demonstrates the potential value to workforce planners of routine data linkages between staffing e-rostering systems and patient data within healthcare organisations.

### Data sharing

The electronic staff rostering data and the individual patient records cannot be shared by the investigators under the data use agreement with the NHS Trust studied in this paper.

## Data Availability

Data may be obtained from a third party and are not publicly available. The electronic staff rostering data and the individual patient records cannot be shared by the investigators under the data use agreement with the National Health Service Trust studied in this paper.
